# Towards the identification of humic ligands associated with iron transport through a salinity gradient

**DOI:** 10.1038/s41598-022-19618-2

**Published:** 2022-09-15

**Authors:** Kavi M. Heerah, Heather E. Reader

**Affiliations:** 1 Environmental Sciences Program, Memorial University of Newfoundland and Labrador, St. John’s, NL Canada; 2 Department of Chemistry, Memorial University of Newfoundland and Labrador, St. John’s, NL Canada

**Keywords:** Biogeochemistry, Carbon cycle, Element cycles, Environmental chemistry, Marine chemistry

## Abstract

Humic ligands from boreal rivers have been identified as important sources of iron-binding ligands to the coastal marine environment but remain poorly characterized. A novel method using Fourier transformed infrared spectroscopy (FTIR) was used to identify and quantify iron-binding ligands present in a boreal river in Newfoundland, Canada. 20 to 35% of the total iron load was carried through an artificial salinity gradient, and remained in solution at 35 salinity. Using FTIR combined with linear regression and 2D correlation analysis, we identified two pools of organic ligands, with different behaviour with regards to iron across the salinity gradient. The weaker ligand pool consisted of alkenes, ethers, and esters, and was found to release iron to flocculation at low salinities, and not contribute to iron transport into the marine environment. The stronger ligand group contained carboxylic acids and aliphatic functional groups. This group appears to contain two subgroups, one which was able to retain iron in the dissolved phase at 35 salinity, and another that flocculated out with iron across the salinity gradient. The strong ligands that retain iron in solution through the salinity gradient provide a much-needed source of the micronutrient to the coastal and marine environment, while the other subgroup sequesters iron and carbon in estuarine sediments. The balance between these two subgroups appears to be controlled by the hydrographic and weather conditions at the time of sampling, suggesting a dynamic ligand-iron relationship throughout the year, impacting the biogeochemical cycles of both iron and carbon in contrasting ways.

## Introduction

### Importance of iron

Iron is a key element influencing ocean productivity and global biogeochemical cycles^1,2^. It is utilized by phytoplankton to carry out nitrogen fixation, photosynthesis, and respiration^1,3^ and has been shown to play a role in carbon sequestration by association with organic matter in sediments, protecting the carbon from microbial degradation and thus enhancing long-term carbon storage^4,5^. The importance of iron on a global scale for biological productivity and global cycles has stimulated multiple studies to better understand sources, utilization, and how changing physico-chemical conditions affect its distribution.

Despite its importance dissolved iron exists at very low concentrations in marine environments due to its low solubility and can be a limiting nutrient for vast areas of the ocean^2^. The major sources of iron to the ocean are thought to be atmospheric deposition and hydrothermal vents^1,2^. Most of the iron found in the marine environment is not free iron, rather the majority of it remains in solution through complexation with organic ligands^6^. These ligands can come from a variety of sources such as siderophores, humic substances, exopolymeric substances, porphyrins, and saccharides^6–8^. Humic ligands, a subset of the so-called “humic substances”, originate in terrestrial environments and in the marine environment, and in some regions constitute a large fraction of the total iron-binding ligand pool^9–13^. Humic ligands, like all dissolved organic matter whether terrestrial or marine in origin, are generally poorly characterized from a molecular standpoint. Terrestrially-derived humic ligands can act as a source of riverine iron to the marine environment, particularly in regions highly influenced by riverine inputs, such as coastal regions^10,13^ and even open ocean regions like the Transpolar Drift Current in the Arctic Ocean, which has been shown to be a major source of terrestrially derived trace metals and organic matter^14,15^.

Despite being a small percentage of total marine iron, riverine iron has recently been recognised as an important source due to its increased bioavailability^9,16–18^.The need to understand the controls and transport of iron plays a key part in better understanding ocean productivity. Riverine iron was previously ignored as a significant source of iron to marine environments, as much of riverine iron is lost through flocculation over the salinity gradient, and subsequently buried in estuarine sediments^19^. Historically, 95% of a river’s iron load was thought to be lost through the estuary but through complexation with humic ligands more recent studies report up to 20% of the total dissolved iron load can be transported through the salinity gradient into the ocean^16,18,19^. An increase in the iron load can disrupt the local marine environment and further, causing brownification and increased productivity^2,20^.

### Humic-iron complexes and their importance

Dissolved organic matter (DOM) is formed from the breakdown of organic matter, leaf litter, organisms’ secretions, etc.^21^. The composition of DOM is a direct reflection of the environment it is found in. DOM from peatlands and boreal environments are enriched in humic ligands supporting an increased iron load. Humic ligands are still poorly understood, in large part due to the chemical complexity of DOM. A number of studies have found that humic ligands are responsible for maintaining the majority of dissolved iron found in coastal environments, especially in the coastal North Atlantic^9,10,13^. While humic ligands are of both terrestrial and marine in origin in most coastal marine systems, the coastal North Atlantic, and particularly the region around Newfoundland, receives large amounts of DOM from rivers draining boreal forest and peatlands, including terrestrial humic ligands.

An understanding of terrestrial humic ligands and their relationship to iron is hindered by the complexity of DOM, but their influence on iron and its speciation in the marine environment has been of recent interest^14,15,17,18,22,23^. Iron concentrations have been increasing in boreal freshwaters over past several decades^24^, and the reasons for these increases have been attributed to a range of environmental factors, such as reduced acidification, changing redox conditions, ionic strength, warming, and hydrology^24–28^. At the same time, dissolved organic carbon (DOC) concentrations have been increasing in the same regions^28–34^, thought to be driven by an equally complex range of factors. Björnerås et al.^24^ point out that though iron and DOC concentration are often rising concurrently, their behaviours are not necessarily directly linked, but rather they may be responding to similar drivers.

In addition to increased iron and DOM loading from these various environmental variables, changing climate conditions are projected to bring more frequent and intense storm events to these regions^35^. Storm and related flooding events cause increased flow regimes within catchments which can affect the sources and therefore quality of DOM entering the coastal system^36–39^. During storm events, different layers of the catchment’s soil can be accessed providing DOM from unique sources. During these events mobilization of a wide variety of substances can occur, causing increases in nutrient, DOM, and metal concentrations. Changes in flow paths through the catchment can increase or decrease the fraction of humic ligands in the DOM and potentially change which specific ligands are present^30,40^.

Regardless of the mechanism, the combined effects of increased iron and DOM export out of boreal rivers has the potential to deliver more dissolved iron to coastal regions in association with terrestrial humic ligands. This has the potential to further increase the importance of the terrestrial fraction of iron-binding humic ligands in the marine environment in the coming years. Krachler et al.^17^ recently demonstrated that terrestrial humic substances had the potential to increase dissolved iron concentrations in the open ocean even at extremely small concentrations, driving home the importance of these ligands.

As a natural substance, DOM exists in a complex matrix that makes it difficult to carry out deep structural analysis on untreated samples^41,42^.There are multiple ways to investigate DOM, but it remains difficult to fully characterise this complex substance. One such tool is Fourier transformed infrared spectroscopy (FTIR). FTIR can be used to identify functional groups present in a sample giving useful structural knowledge without the associated cost and time of other methods such as mass spectroscopy (MS) and nuclear magnetic resonance (NMR)^41^. FTIR has been used to study DOM before, but this is often done in conjunction with other analytical techniques, providing only qualitative data^43^. In this study, we used FTIR to identify functional groups present in DOM from a small boreal peatland catchment. FTIR was used in a quantitative fashion to track the changes functional groups undergo through an artificial salinity gradient as iron is flocculated out. Four samples were collected to capture two high flow regimes (H1 and H2), and two low flow regimes (L1 and L2). The resulting spectra were then analysed by applying perturbation-based 2D-correlation spectroscopy (2D-COS)^44,45^. FTIR was used to address two hypotheses:

1. FTIR can be used in a quantitative method to identify functional groups present in DOM and track how these functional groups change as they travel through an artificial estuary.
2. DOM from a high flow regime will be more enriched in humic functional ligands than those found in base flow.

The use of FTIR in a quantitative method for DOM has not yet been successfully carried out to the best of the author’s knowledge. This study lays out a method for identifying functional groups present in DOM and a quantitative way to track their changes across the salinity gradient. This can help further expand the knowledge of nutrient transport on a global scale.

## Results

### Sample characteristics

Initial measurements of the samples show small differences between the samples (Table 1). Overall, pH appears to be driven by flow, with H1 and H2 being more acidic than L1 and L2, and while DOC was similar for both low flow measurements and H2, H1 had a much higher concentration of DOC. Additionally, *a*_350_ (a proxy for the amount of coloured DOM, i.e. CDOM) was higher in the high flow samples than in the low, following the same pattern as DOC .These differences suggest that high flow regimes drain more variable sources of DOC and greater quantity than the low flow regimes. With both higher a_350_ and DOC, H1 appears to be exceptionally different to the other samples.

**Table 1:** Sample characteristics at the time of sampling.

Sample	Discharge (m^3^/s)	pH	Temp (°C)	*a*_350_	DOC (mmol/L)	Iron (µmol/L)	Date
High Flow 1 (H1)	14.00	5.52	0.6	35.68	0.953	3.85	Dec. 16 2020
High Flow 2 (H2)	15.3	5.51	1.4	22.71	0.621	1.93	Feb. 4 2021
Low Flow 1 (L1)	1.88	6.27	0.8	19.51	0.543	1.85	Jan. 26 2021
Low Flow 2 (L2)	1.71	6.6	1.4	15.70	0.414	1.36	Mar. 17 2021

### Experimental conditions

Each of the 4 samples were split into 8 subsamples, and to each subsample an appropriate amount of artificial sea salt was added such that an artificial salinity gradient was achieved, mimicking the changes in salinity across an estuary from freshwater (0 salinity) to open ocean salinity (35 salinity) in steps of 5. This set up allows for the quantification and characterization of the iron and DOM remaining in solution after salinity-induced flocculation processes occur. We analyzed the dissolved iron and the functional groups in DOM that were associated with iron over the artificial salinity gradient. A detailed description of the experimental set up and analyses can be found in the Methods section.

### Iron quantification

**Figure 1:**
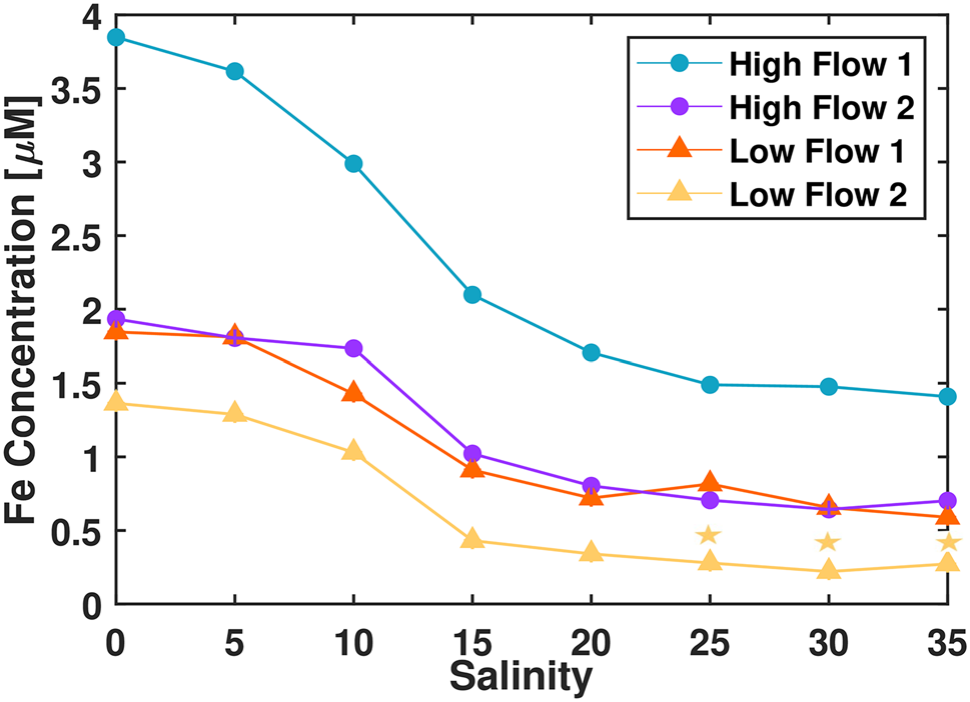
Change in iron concentration (µM) over an artificial salinity gradient. Data collected using a Cary 300 Eclipse spectrophotometer. Data was converted from absorbance to concentration using a linear FeCl_3_ calibration curve. Limit of quantification (LOQ) = 0.28 µmol/L, Limit of detection (LOD) = 0.08 µmol/L. Measurements below LOQ have a small star above them.

Iron concentrations vary between sampling dates with ~3 times more iron in H1 than the other samples (Fig. 1). L1 and H2 are similar with L2 carrying the lowest concentration of iron. All samples experience a pronounced decrease in iron concentration between salinities 10 and 15. Samples continue to lose iron until they reach semi-stable concentrations at salinities 25 to 35. H1(1.41 μmol/L), H2(0.70 μmol/L) and L1(0.59 μmol/L) transport approximately 35% of their total iron load through the salinity gradient while L2 (0.27 μmol/L) carries only 20% of its total iron load at the highest salinities. Initial sample measurements and starting iron concentrations show that H1 samples appear to have a different source of DOM and higher concentrations of mobile iron compared to the other ones. H1 may be more influenced by freshly produced DOM found in the upper layers of peat not accessed during base flow.

### FTIR spectra

**Figure 2:**
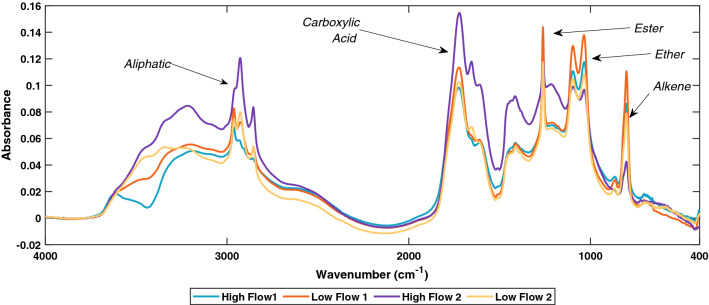
FTIR spectrum of SPE-DOM of the four sampling dates. Samples were measured using KBr pellets and analysed on a Bruker Alpha FTIR. The resolution was at 4 cm/s for a total of 24 scans. Spectra were corrected using a methanol blank and two-point offset correction.

FTIR was used to identify functional groups present in the solid-phase extracted DOM (SPE-DOM) isolated from the salinity gradient experiments. We were able to quantitatively track the changes in the functional groups over the salinity gradient. FTIR spectra from the initial samples (salinity 0) identified five well-defined functional group regions with a sixth unresolved functional group. The functional group peaks identified are; 801 cm^−1^ single peak (alkene), 1097 cm^−1^/1034 cm^−1^ double peak (ether), 1262 cm^−1^ single peak (ester), 1721 cm^−1^ weak triple peak (carboxylic acid), 2963 cm^−1^/2920 cm^−1^/2853 cm^−1^ triple peak (aliphatics), and the sixth unresolved one at 3500 cm^−1^ (Fig. 2).

Of note, H2 appears to be enriched in aliphatic and carboxylic acid functional groups compared to the other samples.

Changes in the FTIR spectra through the salinity gradient can be seen visually in the spectra (Supplementary Fig. S1), and through linear regressions between peak absorbance and iron concentrations (Table 2). These correlations showed that H1, L1 and L2 samples followed similar trends, while H2 exhibited differences in the trends. The aliphatic functional group had negative correlations with iron for H1, L1, and L2, while H2 had a positive correlation. The ester, ether, alkene functional groups had strong negative correlations with iron for H1, L1, and L2, and virtually no correlation for H2. When iron is in solution with these functional groups (at salinity 0), they exist in a complex (i.e., these functional groups represent a class of iron-binding ligands). When bound to iron, the functional groups are inhibited from bending and stretching with IR radiation. As iron precipitates from solution, these ligands remain in the dissolved phase, and no longer inhibited by their association with iron, they are free to absorb IR in their bending and stretching modes, and thus exhibit higher absorbance^46^.While these functional groups are able to associate with iron in freshwater, they are unable to carry the iron through the salinity gradient.

**Table 2:** Linear regression between peak height and iron concentration. Slope and R^2^ are shown for all four dates and the five major functional groups. Regressions were done for each peak meaning functional groups with a double or triple peak have multiple regressions.

Date	High Flow 1		High Flow 2		Low Flow 1		Low Flow 2	
Functional Group	Slope	R^2^	Slope	R^2^	Slope	R^2^	Slope	R^2^
Aliphatic	− 2.66*10^−2^	0.964	1.591*10 ^−2^	0.476	− 3.78*10 ^−2^	0.421	− 3.65*10^−2^	0.857
	− 1.74*10^−2^	0.569	3.011*10 ^−2^	0.481	− 1.23*10 ^−2^	0.082	− 2.03*10 ^−2^	0.493
	− 9.04*10^−3^	0.587	1.916*10 ^−2^	0.509	− 8.08*10 ^−3^	0.082	− 1.23*10^−2^	0.445
Carboxylic Acid	− 1.89* 10^−2^	0.36	3.706*10 ^−2^	0.646	− 1.87* 10 ^−3^	0.001	− 1.35*10^−2^	0.193
Ester	− 6.84*10^−2^	0.835	1.303*10 ^−2^	0.026	− 1.23*10 ^−2^	0.598	− 9.143*10 ^−2^	0.671
Ether	− 6.58*10 ^−2^	0.837	8.046*10 ^−3^	0.008	− 1.21* 10 ^−1^	0.631	− 8.58*10 ^−2^	0.653
	− 6.86*10 ^−2^	0.783	5.705*10^−3^	0.003	− 1.33*10 ^−1^	0.644	− 9.87*10 ^−2^	0.671
Alkene	− 7.23* 10 ^−2^	0.734	− 9.097*10 ^−3^	0.007	− 1.49*10 ^−1^	0.668	− 1.00*10 ^−1^	0.603

The carboxylic acid functional group showed a strong positive correlation with iron for H2, and no correlation for the other samples. The stark contrast between the behaviour of the carboxylic acid peak and the other functional group in all samples shows that the carboxylic acids react differently with iron. The difference in behaviour of the carboxylic acid peak in H1, L1 and L2 compared to H2 further supports a difference in the source and quality of DOM entering the system for H2. The strong positive correlation between the carboxylic acid group and iron in H2 shows co-precipitation of the two species along the salinity gradient. The variability of the high flow sources may lead to different carboxylic acids being exported. In H1, L1, and L2, it is still likely the carboxylic acid is associated with iron, as the acids exist in their charged -COO^-^ form in circumneutral solutions and thus can be strong binding sites for positively charged iron^47,48^, but they do not appear to co-precipitate as in the H2 sample.

The region around 3500 cm^−1^ in IR spectrophotometry is associated with the -OH functional group and also is the region in which the aromatic groups absorb and it follows that important DOM moieties such as phenols would absorb in this region. In our samples, we observed a broad –OH-like peak, and some sharper aromatic-like peaks in this region of the IR spectra. However, despite our best efforts, we were not able to quantifiably distinguish the behaviour of functional groups in this region, due to high variability between samples. This region is also where water strongly absorbs IR radiation and the variability is likely due to moisture absorbed by the KBr during sample preparation and measurement, or even changing humidity in the lab at the time of measurement.

Correlations of peak absorbance and salinity showed the inverse relationship to iron, as expected, with positive correlations where for iron the correlations are negative and vice versa (Supplementary Table S1). The strength of the correlations for individual functional groups and samples did not change greatly between the salinity and the iron regressions.

### 2D correlation spectroscopy

**Figure 3:**
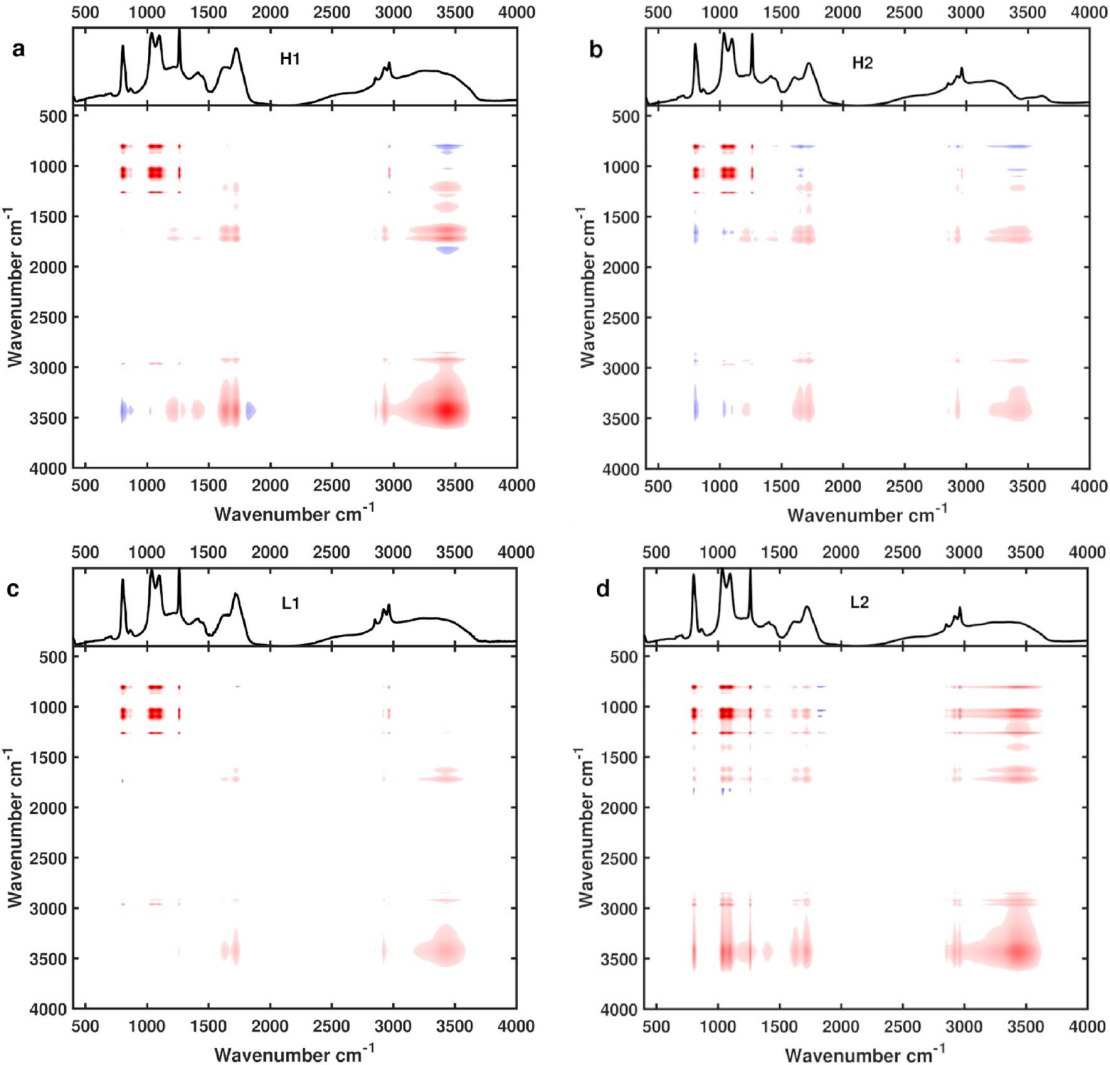
2D COS showing synchronous spectra for the four samples. Spectra were produced following Noda 2016 & 2017. Iron concentration is used as the perturbation with samples up to salinity 25. Panels a, b, c, and d, represent H1, H2, L1, and L2, respectively.

2D-COS is used to identify groupings of individual functional groups, helping to determine which functional groups are part of the same compound. The differences between the carboxylic acid group and the other functional groups is made more evident in the 2D-COS*.* The synchronous spectrum of 2D-COS (Fig. 3) shows how different functional groups change over a perturbation in the system (i.e., change in iron concentration). Functional groups that change in the same direction will develop positive cross-peaks (red in Fig. 3), and those that change in opposite directions will develop in negative cross-peaks (blue in Fig. 3). In all samples strong positive cross-peaks form between the alkene, ether, and ester functional groups. This grouping is supported by the linear regressions showing these three functional groups have similar associations with iron present in the system.

For H1 the carboxylic acid functional group had a weak positive cross peak with the ester, alkene and aliphatic groups. The aliphatic functional group showed weak positive cross peaks with all groups. In L1, the pattern was essentially the same, however the cross peaks that the aliphatic group formed were extremely weak, and nearly non-existent with the carboxylic acid.

A weak negative cross peak developed between the carboxylic acid and the alkene group. In H2, the carboxylic acid and aliphatic were similar and had a positive cross peak between them. Both developed negative cross peaks with the alkene and ether group, with a positive cross peak for the ester. L2 had positive cross peaks develop between all functional groups, with weaker cross peaks being seen for the carboxylic acid group. The strong grouping of the ether, ester and alkene region in the synchronous spectra shows that these groups are related to each other in all samples. The linear regressions further show that they behave similarly for H1, L1 and L2. In H2, these functional groups had low correlations with iron (Table 2), suggesting that in that sample, they are not part of the dominant ligand pool. The difference in behaviour of these groups points to a change in the quality of the DOM in H2 compared to the other samples.

**Figure 4:**
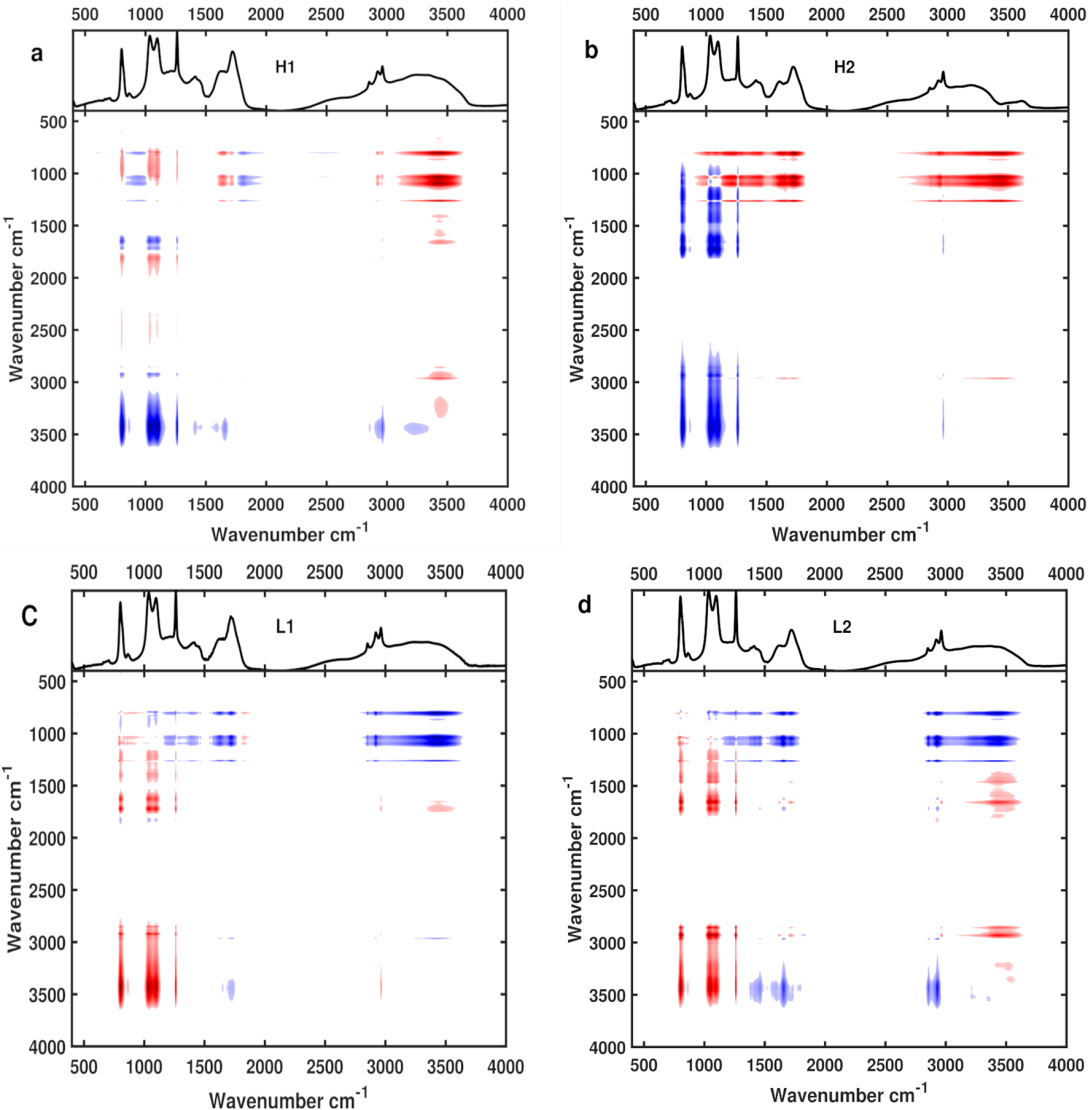
2D COS showing asynchronous spectra for the four samples. Spectra were produced following Noda 2016 & 2017. Iron concentration is used as the perturbation with samples up to salinity 25. Panels a, b, c, and d, represent H1, H2, L1, and L2, respectively.

The asynchronous spectra show if the functional groups are moving in or out of phase with each other, cross-peaks only develop if groups are moving out of phase with each other (regardless of direction). This can help elucidate related functional groups, potentially part of the same compound or group of compounds (Fig. 4). The ether and ester functional groups did not form cross peaks with each other for any flow regime. Interestingly, a small cross-peak between the ether/esters and the alkene groups was observed in H2 and L2, but not H1, and only between the ether and alkene in L1, suggesting that the alkene containing compounds driving the signal are more complex. The carboxylic acid developed strong cross-peaks with the alkene, ether, ester functional groups in all samples. This indicates that the behaviour of the carboxylic acid group is strongly out of phase with the other functional groups, and thus cannot be part of the same compound or group of compounds. Similarly, the aliphatic functional group had strong cross-peaks in relation to the alkene, ether, ester functional groups for all samples.

The relationship between the aliphatic functional group and the carboxylic acid varied between samples. H1 had no cross-peak between these groups, while all of the other samples had very weak cross-peak between the aliphatic and carboxylic acid functional groups, with the strongest cross-peaks found in H2. The variability in relationship between the aliphatic peak and the carboxylic acid group, as well as differences in correlation strength for their respective linear regressions to iron makes it difficult to confidently assign them as being part of the same group of compounds. Though highly degraded DOM is thought to be rich in both carboxylic acids and aliphatics, our samples are enriched in relatively less degraded terrestrial DOM, which may obscure a relationship between the two groups^49^.

## Discussion

### Identification of ligand pools

The aim of our study was to move towards the identification of ligands associated with iron transport from freshwater to marine systems by identifying the specific functional groups associated with iron in a humic rich river.

The ester, ether, and alkene functional groups are clearly part of the same pool of compounds, as can be seen by their strong cross-peaks in the synchronous spectra, and their lack of cross-peaks in the asynchronous spectra. The increase in absorbance as iron is lost from solution indicates that these groups are complexed to iron in freshwater, but the strength of the complexation cannot overcome the tendency for iron to flocculate out of solution at high salinities. The increased freedom for the functional groups to bend and stretch as iron precipitates also confirms that these ligands are staying in solution and no longer associating with iron at higher salinities^46^. Therefore, the ester, ether, and alkene groups belong to a weak ligand class relative to the carboxylate ligands. These functional groups are electron rich with double bonds and oxygen atoms but are not charged (i.e. do not exist as anions), and therefore their association with the positively charged iron cations is relatively easy to reverse as salinity increases.

The strong cross peaks between the carboxylic acid and the other functional groups in the asynchronous spectra show that it is distinct from the other functional groups. Together with the weak correlations in the synchronous spectra and the differences in linear regressions to iron, the carboxylic acids can be determined to be part of a distinct DOM pool from the weak ligand functional groups. Its lack of correlation with iron and salinity in H1, L1, and L2 shows a resistance to flocculation and in these circumneutral waters, carboxylate-iron complex formation is likely. Given the stronger bonding due to the anionic nature of the carboxylates, coupled with potential chelating effects to the positively charged iron, these ligands will be much more challenging to dissociate from the iron, and as such these carboxylate ligands form a strong ligand pool. Carboxylic acids are expected to exist in abundance in the peat dominated soils present in the watershed.

In contrast, H2 exhibits a strong positive correlation between the carboxylic acid functional group and iron concentration and a negative correlation to salinity. This contrasting behavior suggests that this fraction of DOM is co-precipitating with iron, rather than a continuing dissolved association in this sample. The stark difference in the behavior of the carboxylic acid functional group between samples indicates that it potentially represents two subgroups of ligands, both strongly associated to iron, but with one that resists flocculation and the other that is readily flocculated over the salinity gradient.

### Relationship of ligands and iron export to flow

Contrary to our hypothesis, overall discharge does not seem to have a major impact on the quality of iron-binding ligands, or the amount of iron exported to the higher salinities. Although differences are seen in the carbon loading and pH of the high flow samples compared to the low flow, structural differences in the DOM between samples appear to be more tied to the time of sampling relative to the event causing the high discharge. While H1 was expected to be significantly different than the low flow regimes based on the bulk properties of the sample, it showed the same two main pools of iron-binding ligands. On the other hand, H2 was expected to behave similarly to H1 based on the bulk properties of the sample prior to the experiment, and yet it showed the most contrasting behaviour. Kritzberg et al.^18^ found that bulk properties of DOM (optical properties, DOC content etc.) were poor indicators of iron carrying capacity in Swedish rivers, and this appears to hold true in our catchment.

Herzog et al.^40^ found more organically complexed iron during spring high flow events, but that there was no relationship between discharge and speciation. They speculate that the pathways of water drainage in the catchment are what actually drive the differences, rather than simply the amount of discharge. This seems to be similar to our results, though the Seal Cove Brook catchment does not experience a single spring freshet event, but rather a number of snowmelt and rain events over the late winter – spring season. Thus, the divergence in DOM behaviour during high flows may be related to the timing of sampling. H1’s high flow was preceded by heavy rainfall, but was sampled on the falling arm of the peak flow, allowing time for the rainfall to percolate through the whole soil column and allowing material from the layers accessed by baseflow and the upper layers to be exported out. In H2, the high discharge was preceded by a large amount of snowmelt and rainfall, and was sampled just prior to peak flow on the rising arm, while it was still raining heavily. In peat systems, the soil is comprised of different layers which contribute different DOM^36,37,50,51^. These distinct layers are accessed during different flow events and seasons changing the quality of DOM entering the system. By sampling during the rain event, water did not have time to fully percolate into the soils of the catchment and resulted in further reducing the time it comes in contact with the soil prior to entering the river. These factors reduce the proportion of the highly reactive humic DOM from the upper soil layers entering the system^52^ and together with the lower iron concentrations relative to H1, the less reactive DOM in H2 is unable to form the necessary complexes to stabilize both the iron and DOM as it travels through the salinity gradient. While normally these moments of intense flow are expected to stimulate productivity in the coastal environment, these results suggest that for Seal Cove and potentially other parts of Newfoundland, increasing discharge alone is unable to stimulate further productivity without additional factors such as greater infiltration through the soil bed or seasonal factors, tipping the balance towards iron and carbon burial in the estuary during these extreme events.

Despite experiencing variable discharge throughout the year, with no clear seasonal peaks^53^, the catchment itself is more productive during the summer months. Seasonal differences in DOM export have been found in other similar boreal rivers, with the bulk quality of carbon changing over the year, something that would be expected in Seal Cove Brook as well^39^. Indeed, it has been shown that the behaviour of iron export and the balance between inorganic vs organic forms can vary throughout the year in an high-humic estuarine setting^19^. Thus, the seasonality of iron-humic relationships may not only be related to the hydrologic pathways that water takes through the system, but what processes are occurring in the upper layers of soil at the time of sampling. If extreme flow events tip the balance towards iron and ligand co-precipitation year round, they may have less of a stimulating effect on productivity in the marine environment and may partially mitigate the consequences of increasing precipitation in boreal regions. On the other hand, if there is a confounding effect of seasonality in ligand export and more iron remains in solution during extreme precipitations events in the summer productive months, estuarine productivity could be greatly increased. Given the variability of discharge throughout the year in catchments like Seal Cove Brook, the seasonal changes in iron and humic export deserve further investigation to fully delineate the sources and fates of the different ligand groups in the coastal marine environment.

## Conclusion

We were able to identify two classes of iron-binding humic ligands in a small boreal river, using FTIR and an artificial salinity gradient. The weaker ligand class is characterized by ester, ether, and alkene functional groups, and while it is clearly complexed to iron in freshwaters, it readily releases iron to flocculation at estuarine salinities. The stronger ligand class is characterized by carboxylic acid functional groups, and appears to be comprised of two sub-pools, one resistant to flocculation and the other co-precipitating with iron at higher salinities. The first sub-pool that is resistant to flocculation is responsible for transporting the high amounts of iron found in boreal rivers to the coastal ocean, whereas the second sub-pool likely contributes to the sequestration of iron and carbon into the sediments, a process coined “the rusty carbon sink”^5^. The balance between iron remaining in solution aided by organic complexation or co-precipitating with organic carbon appears to be related to the specific hydrology at the time of sampling, and may also have a seasonal aspect. This implies a changing influence of iron and DOM throughout the year, affecting the balance between complexed iron in solution available to the microbial community and preservation of both iron and carbon in estuarine sediments. These two contrasting scenarios have opposing effects on the biogeochemical cycles of both iron and carbon. The implications of seasonal and/or weather driven changes on such contrasting fates of terrestrially sourced iron and DOM in marine environments are significant, and deserve further investigation. The lack of information on the chemical composition of iron-binding ligands has so far severely hindered the understanding the biogeochemical cycle of iron. Through the identification of the functional groups present in terrestrial humic ligands and their differing behaviours over the salinity gradient, this study represents a major step forward towards fully determining the role that terrestrially-derived iron-binding ligands play in determining the fate of iron and organic matter in marine systems.

### Study site and methods

#### Study site

The Seal Cove Brook watershed is a small watershed approximately 56 km^2^ in size. It is located in the hyper-oceanic barrens ecoregion of Newfoundland, Canada. The area is dominated by small lakes (12.87%), peatland barrens (33.63%), forest (50.75%), and wetlands (1.95%) ^37^. Four samples were collected from December 2020 to March 2021, capturing two high discharge rates and two low discharge rates. (Table 1). Discharge rates were obtained through the Environment and Climate Change Canada Real-Time Hydrometric Data for Seal Cove Brook (station number 02ZM009)^53^.

Six liters of water were collected each time from the middle of the river, using a plastic bucket (rinsed several times prior to sampling) and was immediately transferred to acid-washed (cleaned with 10% HCl solution) 2 L polycarbonate bottles (Nalgene, rinsed several times with sample prior to filling). The samples were transported on ice in the dark back to the lab. Samples were filtered through ashed Whatman GF/F filters (0.7 µm, 450 °C for 4 h). Salinity, water temperature, and pH were taken in situ using a Thermo Scientific Orion Star A3229 meter equipped with conductivity and pH probes. Absorbance was taken after filtration, using a Cary 300 series UV–visible spectrophotometer, from 200 to 800 nm, with 1 nm resolution. We calculated the Napierian absorbance coefficient at 350 nm, using the equation ${a}_{350}=2.303A/l$, where A is the absorbance and *l* is the pathlength of the cuvette in meters.

#### Experimental setup

An artificial sea salt mixture was made following the methods of Søndergaard et al.^54^ and Kritzberg et al.^18^. The salt recipe is for 50 g and is as follows: sodium chloride (ACS grade, ACP Chemicals*)* 38.8 g , sodium sulfate (ACS grade, ACP Chemicals*)* 5.8 g*,* and potassium chloride (ACS grade, Fisher Chemical*)* 0.96 g were ashed (450 °C for 4 h) to reduce organic contamination. Magnesium chloride hexahydrate 15.5 g , calcium chloride dihydrate 2.5 g , and sodium bicarbonate (all ACS grade, Fisher Chemical) 0.29 g were used directly to avoid degradation of the salts.

The filtered sample was divided into eight 250 mL glass mixing cylinders. Artificial salt mixture was then added to each cylinder to form a salinity gradient from 0 to 35 ppt in intervals of 5 ppt. Samples were shaken to dissolve the salt and placed on an orbital shaker at 100 rpm for 1 h before being left in the dark to settle for 24 h. The supernatant (150 mL) was gently transferred from the glass cylinders using HDPE syringes (no rubber parts) and gravity filtered through ashed GF/F filters (Whatman, nominal pore size 0.7 µm, 450 °C for 4 h). Subsamples for iron quantification and solid-phase extraction were collected from the filtrate.

#### Iron quantification

Iron quantification was carried out immediately after gravity filtration following the methods outlined by Viollier et al.^55^ and Kritzberg et al.^18^. Briefly, 2.5 mL of sample were heated gently to ~ 90 °C with 515 µL of 1.4 M hydroxylamine hydrochloride (99%, ACROS Organics) prepared in 2 M HCl (ACS grade, ACP chemicals), and 250 µL of 0.01 M FerroZine iron reagent hydrate (98%, ACROS Organics) prepared in 0.01 M ammonium acetate (97%, Alfa Aesar) for 10 min. The sample was then removed from heat and allowed to cool for 90 s before adding 200 µL of 10 M ammonium acetate buffer previously adjusted to pH 10 with ammonium hydroxide (ACS grade, ACP chemicals). The absorbance of the FerroZine-iron complex was measured at 562 nm, using a Cary 300 series UV–visible spectrophotometer (Agilent). A sample blank for each measurement was also carried out to account for any interference from the DOM. To carry out the sample blank, Milli-Q water is used in place of the chemical reagents. The limit of detection (LOD) was determined to be 0.08 µmols/L and the limit of quantification (LOQ) was 0.28 µmols/L.

#### Solid-phase extractions

Solid-phase extractions were carried out using 100 mg PPL cartridges, loading 20 µmols of dissolved organic carbon (DOC) on each cartridge, and following the general procedure described in Dittmar et al.^42^, slightly modified by soaking the cartridges with HPLC grade methanol (Fisher Scientific) overnight to remove any contaminants from the resin prior to extraction. Cartridges were then washed with 100 mL of acidified Milli-Q water (pH 2) at a flow rate of 10–12 mL/min. A reduced flow rate of 4 mL/min was used to pass the sample through. The cartridge was then washed with 30 mL of acidified Milli-Q water (pH 2) at 4 mL/min. The cartridge was dried on vacuum and stored at − 18 °C until it was extracted. Extraction was carried out using 2 mL of methanol at a flow rate < 1 mL/min. The extract was dried using a mini-air evaporator attached to an air pump.

For each gradient, the DOC content of the initial sample was used to determine the volume needed for the extraction. DOC was measured as non-purgeable organic carbon using a Shimadzu TOC-L analyser. Samples were acidified by hand with 2 M HCl. The analyser was calibrated daily with acetanilide (99%, ACROS Organics) standards, and the covariance on each injection was < 2%. An in-house seawater reference and the DOC consensus reference material (Hansell Lab, RSMAS, University of Miami). Due to the high organic carbon concentrations in the samples, only a small volume was needed for each extraction (20–40 mL).

#### FTIR measurement

The dried sample was analysed using a Bruker Alpha FTIR, resolution of 4 cm^−1^ over a range of 400–4000 cm^−1^ for a total of 24 scans. FTIR samples were prepared using KBr (FTIR grade, > 99% trace metal basis, Sigma Aldrich). KBr was dried overnight at 115 °C before being placed in a desiccator. Dried samples were redissolved in 100 µL of HPLC grade methanol before being dropped in 10 µL intervals on an agate mortar, allowing the methanol to evaporate completely. An infrared lamp (Fluker’s Red Heat bulb, 60 Watts) was used to maintain a temperature around 30 °C and prevent moisture from condensing into the sample. Once the sample was dried 200 mg of KBr was ground in the mortar to form a homogenised mixture. The KBr-DOM mixture was placed in a KBr die and pressed at 16 tonnes of pressure in 4 tonnes steps every 20 s. The sample was then immediately analysed on the FTIR in transmittance mode. A methanol blank was carried out before analysing the samples, where 100 µL of methanol was dropped and evaporated on the agate mortar, and the KBr was ground in the mortar in the same fashion as with a sample.

KBr spectra were processed with MATLAB R2020a. All statistical analyses carried out on the samples were only carried out up to salinity 25 or the point where the concentration of iron went below the LOQ. By salinity 25 the change in iron concentration in all samples had reached a semi-stable state and little to no further loss of iron was observed at the higher salinities. Samples were converted to absorbance using $Absorbance= -{log}_{10 }T$, where T is the percent transmittance of the sample. The methanol blank was subtracted from each sample and a two-point offset baseline correction was applied. Peaks were identified using standard FTIR tables^56^. Simple linear regressions were carried out between peak height versus iron concentration and peak height versus salinity.

Following the methodology of Noda^44,45^ 2D correlation spectra (2D-COS) were calculated, giving synchronous spectra and asynchronous spectra. Briefly, a series of IR spectra is exposed to an external perturbation (in this case a change in iron concentration). Carrying out a cross-correlation function produces a synchronous plot with cross-peaks showing co-incidental changes in intensity over the perturbation. Applying a Hilbert-Noda transformation produces a new set of spectra that is 90° out of phase to the referenced spectra^45^. This asynchronous spectrum will only have cross-peaks if the signals are changing out of phase with each other over the perturbation. Combining the information in both spectra allows for the identification of signals resulting from the same and different chemical species within the mixture. Only peaks representing a change of more than 5% of the maximum intensity of cross-peaks are plotted. This cutoff was used to ensure that data interpretation was restricted to changes in chemical functional groups and not baseline shifting between samples.

## Supplementary Information

Supplementary Information.

## Data Availability

The data for this study can be found at Memorial University Borealis data repository. URL: https://borealisdata.ca/privateurl.xhtml?token=ae4dddf1-acb4-49ac-8151-b5d3722c4052.
