# Supplementary material for: Towards the identification of humic ligands associated with iron transport through a salinity gradient
Source: Sci Rep. 2022 Sep 15;12:15545. doi: 10.1038/s41598-022-19618-2 (PMC9477803; doi:10.1038/s41598-022-19618-2)
Supplement: Supplementary file 1 — Supplementary Information. [file 41598_2022_19618_MOESM1_ESM.pdf]

Towards the identification of humic ligands associated with iron transport through a salinity gradient

Kavi Heerah and Heather Reader

Page 2: Figure S1: Change in FTIR spectrum over a full salinity gradient for all four samples.

Page 3: Table S1: Linear regressions of peak height vs salinity for the five functional groups identified

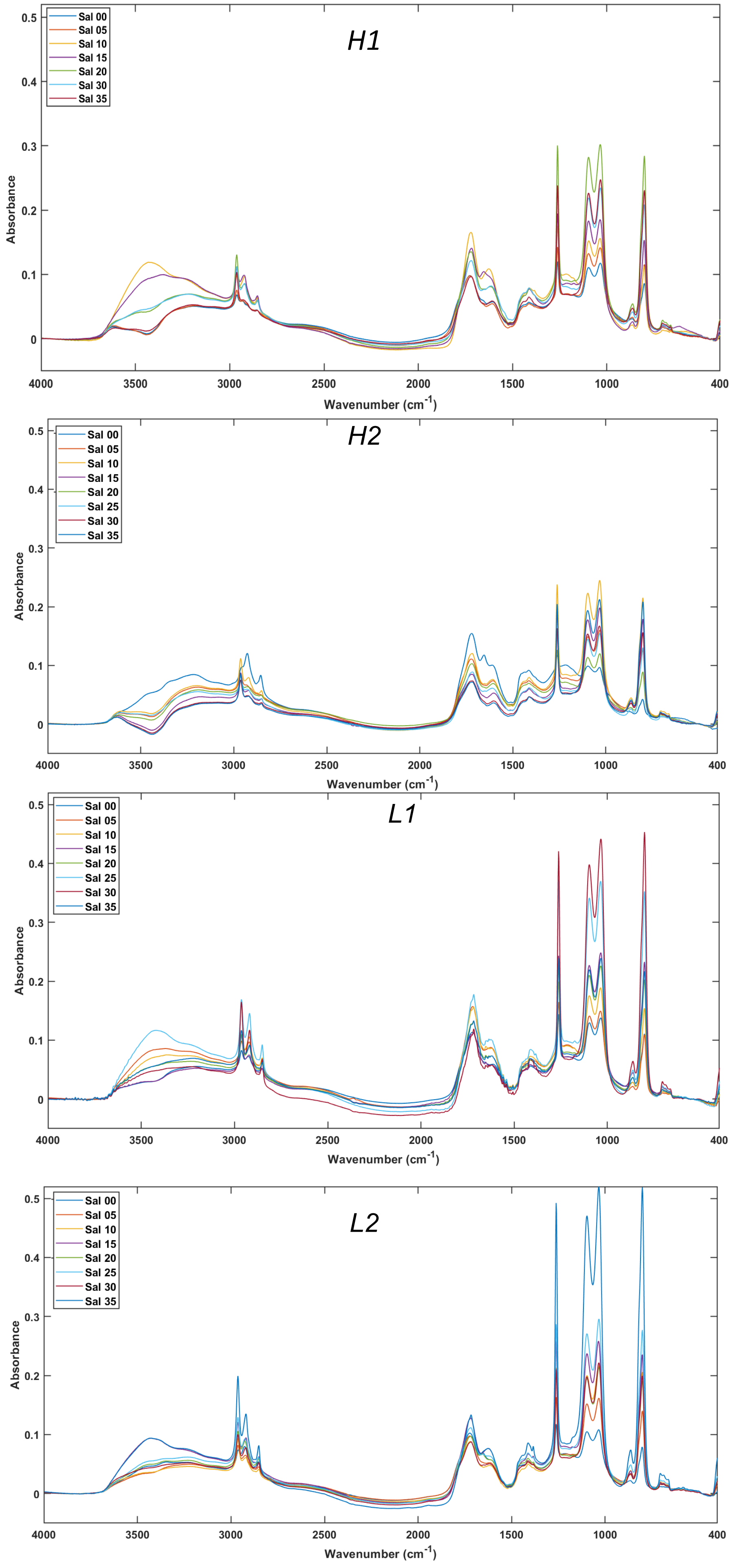

Figure S1: Change in FTIR spectrum over a full salinity gradient for all four samples. Samples were collected on a Bruker Alpha FTIR spectrometer in the form of KBr pellets at a resolution of 4cm^{-1} over a range of 4000-400 cm^{-1} for a total of 24 scans. Spectra was corrected using a 2 point offset correction and a methanol blank . December is missing salinity 25 due to contamination issues

Table S1: Linear regressions of the five functional groups identified comparing peak height to salinity. All regressions carried out in MATLAB 2020A

**Date**	**H1**		**L1**		**H2**		**L2**	
Functional group	Slope	R^2	Slope	R^2	Slope	R^2	Slope	R^2
	$3.16*10^{-3}$	0.979	$2.68*10^{-3}$	0.720	$-7.13*10^{-4}$	0.303	$2.07*10^{-3}$	0.75
Aliphatic	$2.05*10^{-3}$	0.57	$1.37*10^{-3}$	0.341	$-1.90*10^{-3}$	0.544	$8.74*10^{-4}$	0.25
	$1.05*10^{-3}$	0.568	$8.91*10^{-4}$	0.341	$-1.21*10^{-3}$	0.59	$5.18*10^{-4}$	0.215
Carboxylic Acid	$2.35*10^{-3}$	0.401	$9.62*10^{-4}$	0.128	$-2.22*10^{-3}$	0.668	$3.82*10^{-4}$	0.042
Ester	$8.26* 10^{-3}$	0.873	$7.86* 10^{-3}$	0.825	$1.12*10^{-4}$	$6.34*10^{-4}$	$5.58*10^{-3}$	0.682
Ether	$7.88* 10^{-3}$	0.862	$7.53*10^{-3}$	0.833	$4.41*10^{-4}$	0.008	$5.32* 10^{-3}$	0.685
	$8.24*10^{-3}$	0.814	$8.23* 10^{-3}$	0.835	$7.05* 10^{-4}$	0.015	$6.14*10^{-3}$	0.709
Alkene	$8.68*10^{-3}$	0.759	$8.97*10^{-3}$	0.828	$1.66* 10^{-3}$	0.063	$6.40*10^{-3}$	0.672
